# Data showing the effects of disc milling time on the composition and morphological transformation of (α+β) titanium alloy (Ti–6Al–2Sn–2Mo–2Cr–2Zr-0.25Si) grade

**DOI:** 10.1016/j.dib.2019.104174

**Published:** 2019-06-24

**Authors:** Okwudili Simeon Ogbonna, Stephen A. Akinlabi, Nkosinathi Madushele

**Affiliations:** a Department of Mechanical Engineering Science, Auckland Park Kingsway Campus, Faculty of Engineering and Built Environment, University of Johannesburg, 2006, South Africa; b Department of Mechanical and Industrial Engineering Technology, Doornfontein Campus, Faculty of Engineering and Built Environment, University of Johannesburg, 2006, South Africa

**Keywords:** Titanium alloy, Milling time, SEM, EDS

## Abstract

In powder metallurgy, dry mechanical milling process is an effective technique employed in the reduction of solid materials into the desired size in the fabrication of materials or components from metal powders for various applications. However, the milling operation introduces changes in the size and shape as well as the elemental or chemical composition of the milled substance. These changes introduced after milling requires critical analyses as the performance and efficiency of fabricated components depend so much on the size, shape and chemical composition of the powders. In this data, the effects of vibratory disc milling on the morphological transformation and elemental composition of titanium alloy powder were observed and analyzed by Scanning Electron Microscopy (SEM) and Energy Dispersive Spectroscopy (EDS). The as received titanium alloy powder was subjected to dry mechanical milling machine rated 380V/50Hz at 940 rpm. Milling time of 2, 4, 6, 8 and 10 mins were adopted in this data collection. SEM and EDS analyses revealed that milling transformed the spherical shaped powders into plate-like shapes. This deformation in the shape of the powder increased with increase in milling time. Also, the oxygen content of the powder fluctuated as the milling time increased.

**Table:** Specifications table

Subject area	Mechanical Engineering and Material Science
More specific subject area	Powder Metallurgy
Type of data	Micrographs and table
How data was acquired	The titanium alloy powder was milled by digital vibratory disc milling machine, Model 2MZ-200 at different milling time of 0, 2, 4, 6, 8 and 10 minutes. The powders obtained after each milling time were characterized by scanning electron microscopy (SEM), Tescan VEGA 3 LMH type equipped with energy dispersive spectroscopy (EDS) operated by oxford software.
Data format	Raw and analyzed
Experimental factors	There was no pretreatment of the powder. It was then milled at 0, 2, 4, 6, 8 and 10 mins with vibratory disc milling machine.
Experimental features	The powders collected at each milling interval together with the as received powder were analyzed by SEM micrograph equipped with EDS operated by oxford software.
Data source location	University of Johannesburg, South Africa.
Data accessibility	Data is with this article.

**Table:** 

**Value of the data** • The data give an idea of the mechanism of deformation involved in mechanical milling of metal alloy [1], [2]. • These data are useful for manufacturers of particle reinforced composite materials. • The data give an insight of milling time that might be adopted depending on the desired particle size. • These data are relevant to researchers dealing with metal powders for further studies on mechanical milling operations.

## 1 Data

**Fig. 1:**
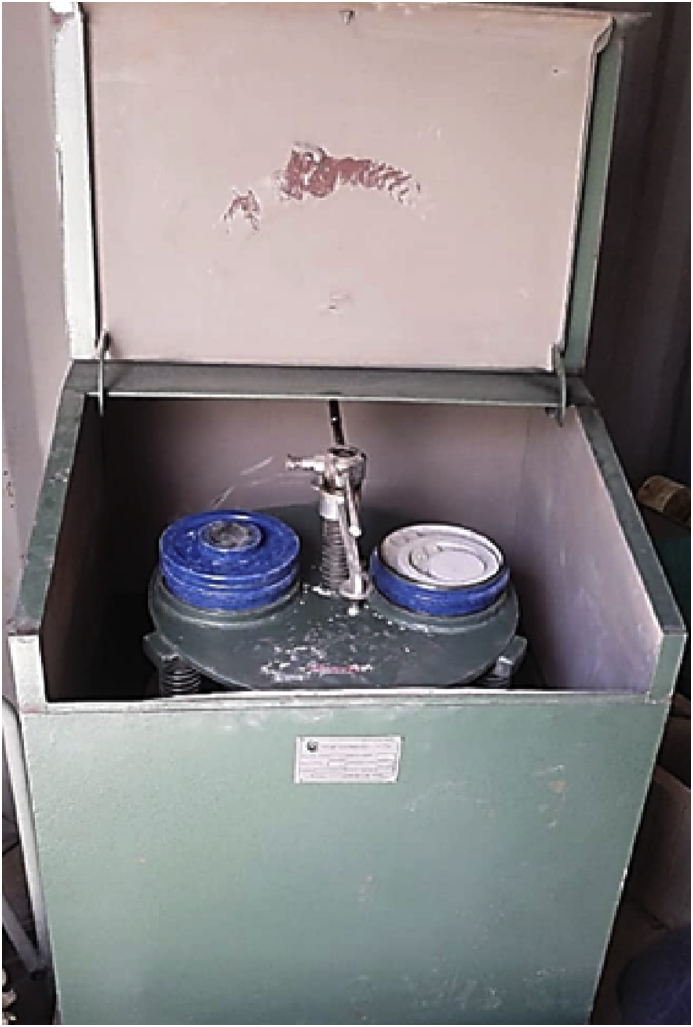
Double chambered vibratory.

The data presented here are the scanning electron microscopic (SEM) images and energy dispersive spectroscopy (EDS) of titanium alloy powder presented in Fig. 3, Fig. 4 milled by digital vibratory disc milling machine as shown in Fig. 1 indicating the effect of milling time on the morphological transformation and elemental composition as seen in Table 2.

## 2 Experimental design, materials and methods

The material used for this dataset is titanium alloy powder (Ti–6Al–2Sn–2Mo–2Cr-0.25Si with particle size of 45–90 μm) manufactured and supplied by TLS Technik GmbH & Co company, South Africa. Mechanical milling of the powder was carried out on vibratory disc milling machine at 2 minutes intervals from 2 to 10 minutes. The disc milling machine is shown in Fig. 1.

### 2.1 Experimental methodology

The milling operation was carried out on digital vibratory disc milling machine, Model 2MZ-200 at different milling time of 0, 2, 4, 6, 8 and 10 minutes. Before milling, the milling chamber was washed and cleaned with acetone to eliminate any contaminant. About 40 g of the powder was fed into the milling chamber to keep the experiment running. The machine was stopped at every 2 minutes intervals to set aside the milled powder. The specifications of the machine are presented in Table 1. The powder obtained were then analyzed by scanning electron microscopy and energy dispersive spectroscopy.

**Table 1:** Vibratory disc milling machine specification [3].

Property	Specification
Dimension	740 × 740 × 950 mm
Number of bowls	2
Capacity per bowl	200 g
Feed Size	<15 mm
Motor	380V/50Hz, 1.5KW
Motor speed	940 rpm

**Table 2:** Elemental Compositions of the titanium alloy by EDS.

POWDER	ELEMENT	0 mins Unmilled (%)	2 mins Milled (%)	4 mins Milled (%)	6 mins Milled (%)	8 mins Milled (%)	10 mins Milled (%)
Titanium alloy	Titanium	76.7	81.5	76.3	70.1	66.3	88.8
	Oxygen	16.3	12.1	16.7	14.9	15.1	3.4
	Aluminum	4.9	3.3	4.7	3.8	3.3	5.6
	Carbon	–	0.9	–	7.6	12.2	–
	Zirconium	1.2	1.1	1.3	1.2	1.0	1.5
	Vanadium	0.3	0.4	0.3	–	–	0.4
	Molybdenium	–	–	–	1.9	1.7	–
	Silicon	0.6	0.6	0.6	0.5	0.4	0.3

### 2.2 Scanning electron microscope

**Fig. 2:**
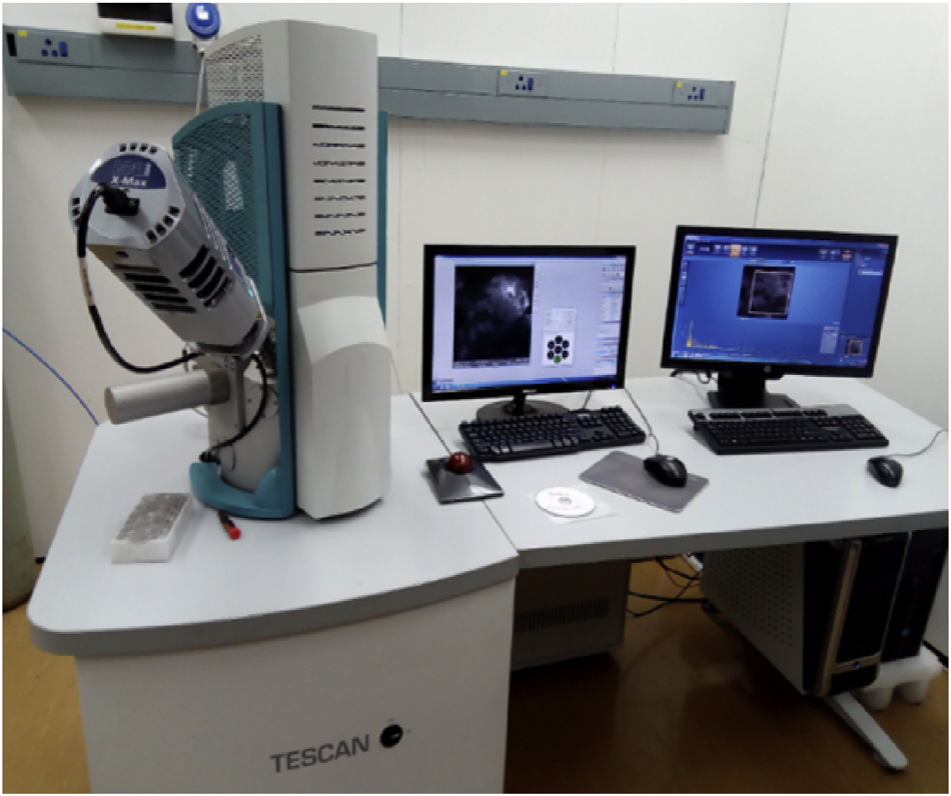
Scanning Electron Microscopy Machine [3] disc milling machine.

**Fig. 3:**
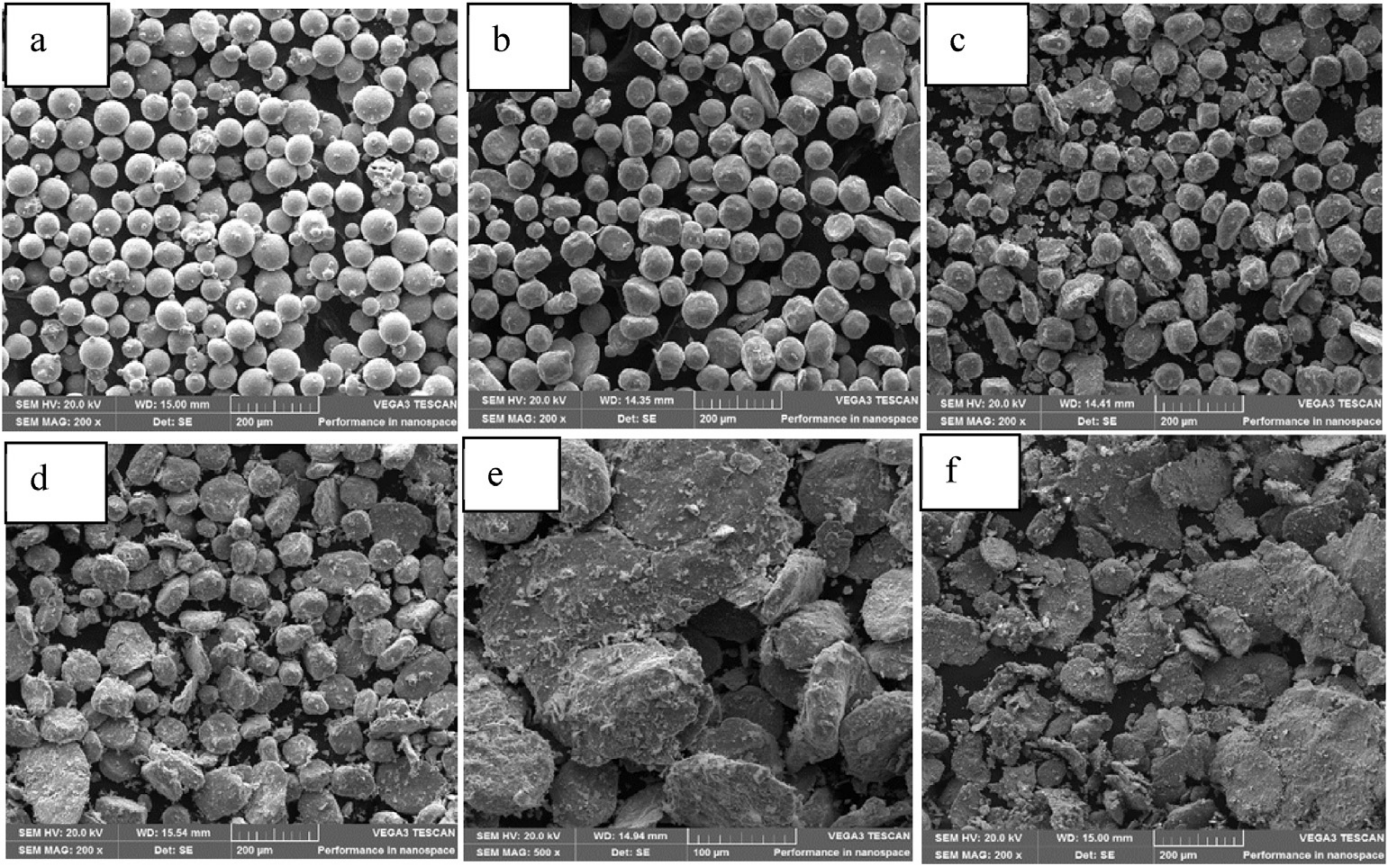
SEM images for the (α+β) titanium powder grade: (a) unmilled – as received, (b) 2 mins milled, (c) 4 mins milled, (d) 6 mins milled, (e) 8 mins milled, (f) 10 mins milled.

The morphological analysis was carried out with scanning electron microscopy, Tescan VEGA 3 LMH type that is equipped with energy dispersive spectroscopy operated by oxford software as shown in Fig. 2. A voltage of 20 KV was selected with a working distance of 15 mm between the specimen and the detector. The scanning electron microscopic images of the milled titanium powder are shown in Fig. 3(a–f).

**Fig. 4:**
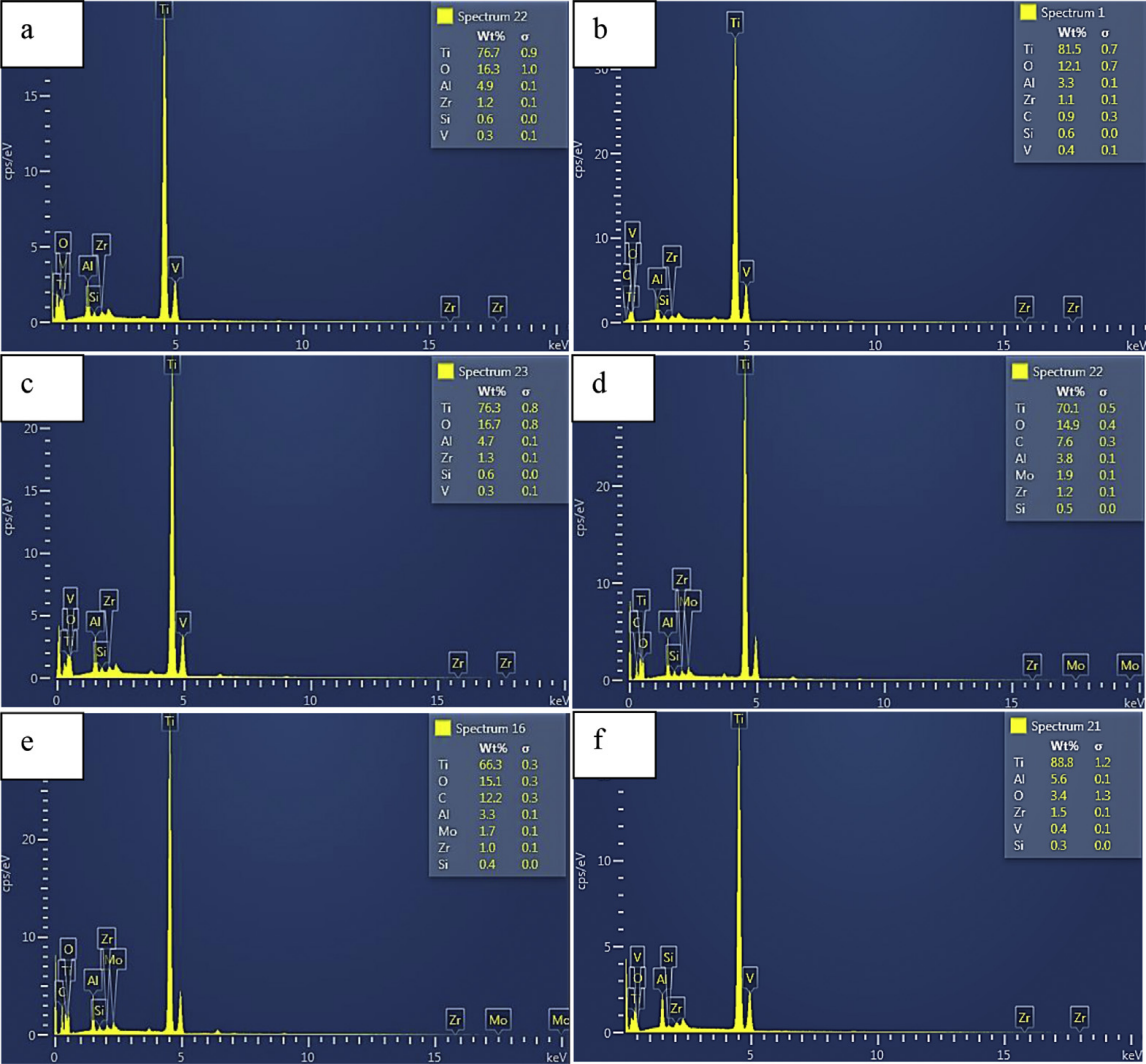
(a–f): Energy dispersive images of the milled titanium alloy: (a) unmilled, (b) 2 mins milled, (c) 4 mins milled, (d) 6 mins milled, (e) 8 mins milled, (f) 10 mins milled.

From Fig. 3, the disc milling operation transformed the spherical titanium alloy powders into plate-like shapes. This also reveals that the ductile nature of the powder.

### 2.3 Energy dispersive spectroscopy

Fig. 4(a–f), show the elemental compositions of the titanium alloy powder as revealed by the EDS images of the milled titanium powder. From the images, the following elements: titanium, oxygen, carbon, aluminum, molybdenum, vanadium, zirconium and silicon were observed. The variation of the elements is presented in Table 2. All the elements showed different percentage content after each milling time. It can be observed that the titanium as the base element of the alloy has the highest percentage in all the cases. Among the alloying elements, silicon as can be observed from Table 2 was the most stable followed by zirconium in terms of percentage variation. Oxygen element was present in all the powder but there was fluctuation in its content with an increase in milling time.

Data Areas of Application.

i) Titanium alloy powder can be used in powder metallurgy for spark plasma sintering [2], [4].
ii) Titanium alloy powder can also be utilized as reinforcement in composite fabrication [5].
iii) Nanoparticles of titanium can be used in the development of nano-lubricants to improve tribological properties [6], [7].
iv) It can also be applied in the improvement of coatings for automobile applications [8], [9].

## Conflict of interest

The authors declare that they have no known competing financial interests or personal relationships that could have appeared to influence the work reported in this paper.
